# EOGT Correlated With Immune Infiltration: A Candidate Prognostic Biomarker for Hepatocellular Carcinoma

**DOI:** 10.3389/fimmu.2021.780509

**Published:** 2022-01-05

**Authors:** Yang Shu, Lingling He, Meixin Gao, Fan Xiao, Junru Yang, Shiwei Wang, Herui Wei, Fuyang Zhang, Hongshan Wei

**Affiliations:** Department of Gastroenterology, Beijing Ditan Hospital, Capital Medical University, Beijing, China

**Keywords:** EGF domain-specific O-linked N-acetylglucosamine transferase, O-GlcNAcylation, immune infiltration, hepatocellular carcinoma, immunosuppressive tumor microenvironment

## Abstract

**Background:**

A preliminary study by our group revealed that the deficiency of EGF domain-specific O-linked N-acetylglucosamine transferase (EOGT) impaired regulatory T-cell differentiation in autoimmune hepatitis. Nevertheless, the prognostic value of EOGT in advanced hepatocellular carcinoma (HCC) and its relationship with immune infiltration remain obscured.

**Methods:**

Initially, EOGT expression was evaluated by Oncomine, TIMER, GEO, and UALCAN databases. Besides, the prognostic potential of EOGT expression was analyzed using GEPIA, Kaplan–Meier plotter, CPTAC, Cox regression, and nomogram in HCC samples. Furthermore, we investigated the association between EOGT expression and tumor mutation burden, DNA methylation, and immune infiltration in addition to its possible mechanism *via* cBioPortal, TIMER, GEPIA, ESTIMATE, CIBERSORT, GSEA, STRING, and Cytoscape.

**Results:**

The expression of EOGT in HCC was significantly higher than that in normal tissues. Additionally, elevated EOGT expression was correlated with advanced tumor staging and linked to poor overall survival and relapse-free survival, serving as a significant unfavorable prognostic indicator in HCC patients. Remarkably, our results revealed that high-EOGT expression subgroups with elevated *TP53* or low *CTNNB1* mutations have worse clinical outcomes than the others. Regarding immune infiltration, immunofluorescent staining showed that immune cells in HCC were positive for EOGT. Besides, elevated EOGT expression was linked to exhausted T cells and immune suppressor cells in HCC samples. More importantly, the proportion of CD8^+^ T cells was reduced in HCC samples with a high level of EOGT expression, but EOGT did not exhibit prognostic potential in HCC samples with increased CD8^+^ T cells.

**Conclusions:**

EOGT may hold great potential as a novel biomarker to distinguish prognosis and immune profiles of HCC patients.

## Introduction

Reportedly, hepatocellular carcinoma (HCC) is the most frequent primary liver malignancy and the fourth most common cause of cancer-related death worldwide (1). Currently, the application of immunotherapy has significantly improved overall survival (OS) and the quality of life, particularly for patients with advanced HCC (2). However, observations from both clinical and preclinical studies have indicated the highly immunosuppressive tumor microenvironment (TME) together with the impaired recruitment of effector T cells in advanced HCC, resulting in limited response rate and resistance to immune checkpoint inhibitors (ICIs) (3). Hence, it is urgent to solve a major unmet need that is limited biomarkers correlated with immunosuppressive TME for patients at advanced stages of HCC.

Protein glycosylation is one of the most well-known post-translational modifications that regulate a large quantity of necessary cellular processes. Currently, accumulating lines of evidence suggested that alterations in glycosylation affect the reciprocal cross-talk between tumor and its microenvironment among various cancer types, including HCC (4). Extracellular N-acetylglucosamine linked to Ser or Thr (O-GlcNAc) is a particular post-translational modification limited to the epidermal growth factor (EGF) domain-containing glycoproteins. EGF domain-specific O-GlcNAc transferase (EOGT) is an endoplasmic reticulum-specific enzyme, which transfers an O-GlcNAc moiety to a restricted number of secreted or membrane proteins, including Notch receptors and ligands (5). In mammals, *EOGT* is one of the disease-causing genes of Adams–Oliver syndrome, an autosomal-recessive disorder (6). Besides, some studies have indicated that alterations in Notch signaling significantly hampered retinal vascular development in *EOGT* mutant mice, which demonstrated that Notch receptors with the mere loss of O-GlcNAcylation have decreased canonical Notch signaling (7). A previous research by our group demonstrated that in *EOGT*-deficient rats, regulatory T-cell (Treg) differentiation was dramatically impaired because of inactivation of Notch signaling, giving rise to abnormal T-cell infiltration into the liver (8). Recently, it was reported that dysregulated Notch signaling mediated by EOGT and lunatic fringe correlated with unfavorable prognosis in pancreatic ductal adenocarcinoma patients (9). Meanwhile, studies had also indicated that Shc SH2-domain-binding protein 1 and EOGT were involved in the progression of pancreatic cancer, promoting O-GlcNAcylation of NOTCH1 (10). Nevertheless, the underlying influences of EOGT in HCC development and potential molecular mechanisms remain unknown.

In the present study, we integrated various bioinformatics methods to focus on whether EOGT is involved in HCC prognosis. Combining with immunofluorescence (IF) staining, we investigated the potential role of EOGT in the progression of HCC. Additionally, we performed EOGT-related gene (ERG) networks and evaluated their biological functions. Moreover, we identified the molecular alterations and immune profile of EOGT and evaluated its effect on clinical outcomes. The results emphasized that EOGT may be a prognostic biomarker as well as an immunological target for the future selection of patients with ICI-responsive HCC.

## Methods

### Analysis of the Relationship Between EOGT and Prognosis

Transcriptome RNA-sequencing data, including 371 HCC samples and 50 normal liver samples, somatic mutation profile of 356 HCC samples, and corresponding clinical information were downloaded from The Cancer Genome Atlas (TCGA) database (https://portal.gdc.cancer.gov/). EOGT expression datasets were extracted for subsequent analyses using R software (Version 4.1.0).

EOGT expression in various cancer types was investigated in Oncomine gene expression array datasets (www.oncomine.org), UALCAN (http://ualcan.path.uab.edu/), Tumor Immune Estimation Resource (TIMER) database (http://timer.cistrome.org/), and The Gene Expression Omnibus (GEO) dataset (https://www.ncbi.nlm.nih.gov/geo/). To explore its prognostic potential in tumor progression, survival analysis was investigated according to EOGT mRNA expression in 12 different cancer types through Gene Expression Profiling Interactive Analysis (GEPIA) (http://gepia2.cancer-pku.cn/).

To confirm EOGT expression at the protein level, immunohistochemical (IHC) analysis of EOGT in normal samples and tumor samples was downloaded from the web of the Human Protein Atlas (HPA) (http://www.proteinatlas.org/) and examined. Plus, Kaplan–Meier (KM) analysis according to the level of EOGT protein expression in HCC samples was performed through Clinical Proteomic Tumor Analysis Consortium (CPTAC) consisting of 151 HCC samples (https://cptac-data-portal.georgetown.edu/). Additionally, cutoff values for survival analysis (mRNA and protein level) were determined by the web tool “auto select best cutoff” in KM plotter (http://kmplot.com/). Proteomic data of EOGT from CPTAC (≤0.05746757 divided into low-EOGT subgroup; >0.05746757 divided into high-EOGT subgroup) were computed to choose the cutoff value in KM analysis.

To further validate the prognostic value of EOGT in HCC, three datasets (GSE54236, GSE76427, and GSE14520) with clinical data were integrated as an external validation set. The gene expression profiles from GSE54236 (including 80 normal liver samples and 81 HCC samples; platform: GPL6480), GSE76427 (including 52 normal liver samples and 115 HCC samples; platform: GPL10558), and GSE14520 (including 241 normal liver samples and 247 HCC samples; platform: GPL571, GPL3921) were obtained from the GEO database. Log2 transformation was performed and batch effects were removed by using the R package “sva.” The average RNA expression value was used when duplicated data were found.

Afterward, to assess the diagnostic value of EOGT in HCC samples, receiver operating characteristic (ROC) curve was performed and the area under the curve (AUC) was calculated. In addition, the prognosis was analyzed by the Cox regression model. Meanwhile, the prognostic nomograms were constructed based on the multivariate Cox model. Through assessment using the concordance index (C-index) and calibration curves, predictive accuracy and discriminative capability of nomograms were effectively quantified.

### Analysis of EOGT-Interacting Genes and Proteins

RNA-sequencing data of 371 tumor samples and 50 normal samples were downloaded from TCGA. Differential expression genes (DEGs) were calculated by the R package “edgeR.” The cutoff criteria of DEGs were *P*-value <0.05 and |logFC| >1. Correlations were examined with Spearman correlation between DEGs and EOGT. *P*-value <0.01 as well as Spearman correlation coefficient (absolute value) >0.3 was defined as ERGs. STRING (http://string-db.org) is an online website dedicated to protein–protein interactions (PPIs). First of all, ERGs were inputted into the STRING database to analyze their interactions. Secondly, isolated protein nodes with no observed connections were eliminated with a minimum required interaction score of 0.4 (medium confidence). Next, PPI pairs were uploaded to Cytoscape software (http://www.cytoscape.org) to display a network and the top 10 hub genes were selected in terms of cytoHubba plug-in of Cytoscape.

### Annotation of EOGT-Related Genes

To investigate the effect of the ERGs on various biological functions, Gene Ontology (GO), Kyoto Encyclopedia of Genes and Genomes (KEGG), and Gene Set Enrichment Analysis (GSEA) were used to predict related biological pathways and molecular function terms in HCC. GO analysis is a useful bioinformatics approach consisting of biological processes (BPs), cellular components (CCs), and molecular functions (MFs). GO and KEGG analyses were performed using the R package “ClusterProfiler”. Then, GSEA was carried out using the GSEA software (http://www.broadinstitute.org/gsea).

### Correlations of EOGT With Molecular and Immune Characteristics

DNA methylation is one of the most common epigenetic events and plays important roles in regulating gene expression, making a great difference in the biological behaviors of tumors (11). EOGT DNA methylation in TCGA-HCC was examined using cBioPortal (http://www.cbioportal.org/). Correlation between EOGT expression and EOGT DNA methylation (HM450) in HCC samples was analyzed. Survival analysis based on EOGT DNA methylation level was explored using KM survival curves, including OS and relapse-free survival (RFS). In the genomic alterations analysis, the number of mutations was evaluated between high- and low-EOGT expression subgroups using the R package “Maftools”. Tumor mutation burden (TMB) is a promising biomarker for ICIs that represents the number of mutations contained in tumor cells. Correlation analysis was also performed between EOGT expression and TMB.

TIMER is a comprehensive database for the systematic exploring of molecular characteristics of tumor-immune interactions. Firstly, co-expression analysis was performed for EOGT and immune-related genes (IRGs) *via* TIMER. Furthermore, GEPIA was also applied to explore the association between EOGT expression and various immune cell surface markers. Moreover, Spearman correlation analysis was used to determine the correlations between the expression of EOGT with T-cell exhaustion and immune suppressor cells in HCC samples. In addition, the HCC cohort from the KM plotter was divided into enriched and decreased subgroups based on mRNA expression of biomarkers of many immune cells in HCC samples. We then investigated the clinical significance of immune cell content in HCC samples.

To further validate immune signatures of HCC samples, the Estimation of Stromal and Immune Cells in Malignant Tumor Tissues Using Expression Data (ESTIMATE) algorithm was applied to utilize existing gene expression profiles to calculate the proportion of stromal and immune cells. The levels of immune infiltration were quantified using immune score and stromal score. Furthermore, we performed CIBERSORT (https://cibersort.stanford.edu/), a computational inference tool, which was used to calculate immune cell composition from gene expression profiles of 547 marker genes. Our study characterized tumor-infiltrating immune cells in HCC samples *via* CIBERSORT and evaluated the association between EOGT expression and 22 immune cell types. Moreover, EOGT expression was further evaluated for different immune cell subsets in HCC using single-cell RNA-sequencing results of six HCC patients from publicly available repository (12).

### Immunofluorescence Staining

To examine the correlations of EOGT, CD31, and EOGT normalized by CD31 (EOGT/CD31) with tumor staging at the protein level, double-labeling IF staining was performed on human tumor microarray (TMA) section for EOGT and CD31. Human TMA slide, containing 67 HCC tissues and 72 normal liver tissues, was purchased from Shanghai Outdo Biotech Co. Ltd (Shanghai, China). Anti-EOGT antibodies (PA5-53990, 1:100) were purchased from Thermo Fisher Scientific. Antibodies to CD31 (GB113151, 1:1,000) were purchased from Servicebio. Double-labeling IF staining was carried out by Servicebio (http://servicebio.com). NIH ImageJ software was used to quantify the intensity of fluorescence.

In addition, to address whether immune cells in tumors are positive or negative for EOGT, double-labeling IF staining was performed on HCC tissue sections for EOGT and CD4, EOGT and CD8, EOGT and CD19, EOGT and CD56, EOGT and CD68, and EOGT and CD11c. Antibodies to CD4 (GB13064-1), CD8 (GB13068), CD19 (GB11061), CD11c (GB11059), CD56 (GB112621), CD68 (GB113150), and CD31 (GB113151) were purchased from Servicebio. Human HCC tissues were obtained from patients with HCC who underwent surgical resection in Beijing Ditan Hospital of Capital Medical University. Patient tissues were collected after obtaining written informed consent. The study was approved by the Ethics Committee of Beijing Ditan Hospital of Capital Medical University for the protection of human subjects. Double-labeling IF staining was carried out by Servicebio. A confocal microscope was used to visualize the cells.

### Statistical Analysis

TPM (transcripts per million) values were normalized by log2 transformation (1+TPM). Student’s *t*-test was performed in the comparison of two groups. Statistical significance was indicated when *P <*0.05. ROC curve was drawn and AUC was calculated by the R package “ROCR”. For survival study, the KM method, log-rank test, and Cox regression were applied. All statistical analyses were implemented with R software (Version 4.1.0). Spearman correlation analysis was applied to statistically evaluate the correlation between two variables. *P <*0.05 indicated significant difference.

## Results

### Pan−Cancer Analysis of EOGT

**Figure 1:**
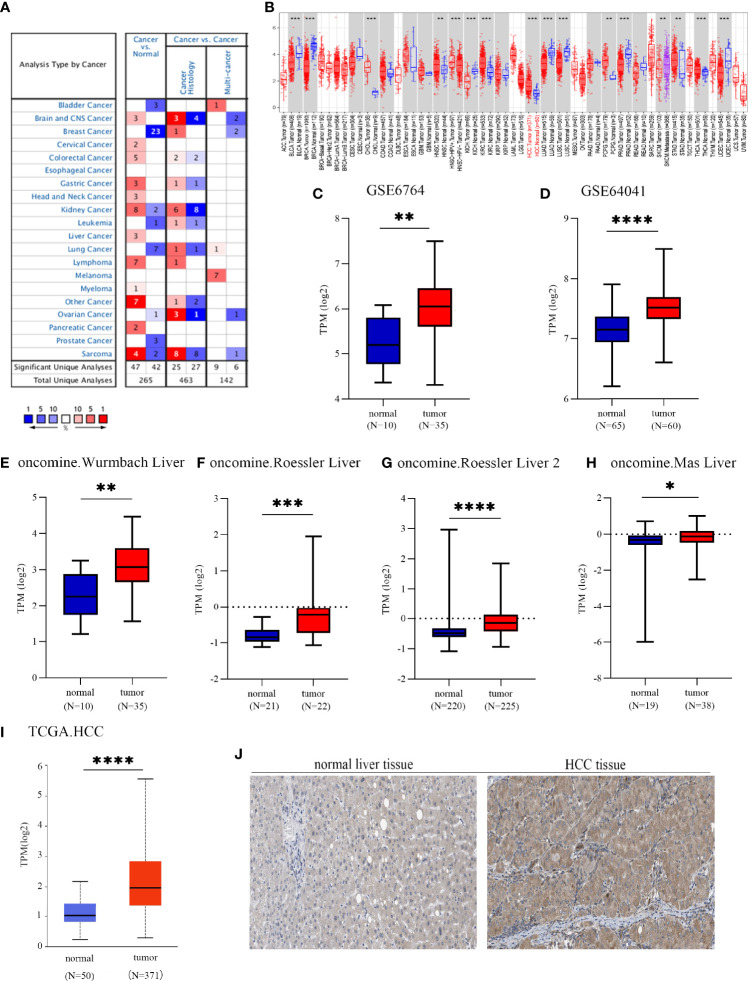
EGF domain-specific O-linked N-acetylglucosamine transferase (EOGT) expression in different types of tumor tissues and normal tissues. **(A)** RNA expression levels of EOGT in different types of tumor tissues and normal tissues in the Oncomine database (*P*-value < 0.01, fold change < 1.5, and gene ranking of all). **(B)** RNA expression levels of EOGT in different types of tumor tissues and normal tissues in the TIMER database. GEO database series including GSE6764 **(C)** and GSE64041 **(D)** and the Oncomine database sets including the Wurmbach liver **(E)**, Roessler liver **(F)**, Roessler liver 2 **(G)**, and Mas liver **(H)**. **(I)** RNA expression levels of EOGT between tumor and normal tissues in hepatocellular carcinoma (HCC) samples on the TCGA database. **(J)** Immunohistochemical staining of EOGT in normal liver tissues and HCC tissues (*P < 0.05, **P < 0.01, ***P < 0.001, ****P < 0.0001).

To investigate the possible roles of EOGT in carcinogenesis, we initially evaluated EOGT mRNA expression in tumors and normal tissues utilizing Oncomine. Pan-cancer analysis of EOGT expression revealed that the expression of EOGT was elevated relative to normal tissues in lymphoma, brain, gastric, cervical, colorectal, liver, head and neck, kidney, and pancreatic cancers. We also found that EOGT expression was lower in bladder, breast, kidney, lung, ovarian, and prostate cancer tissues than in normal tissues (**Figure 1A**). Next, the difference in EOGT expression between tumor and normal samples was detected using TIMER and UALCAN. Data from the TIMER database revealed that EOGT expression was upregulated in seven cancer types, including HCC, but it showed a pattern of decreasing expression in the other seven cancer types (**Figure 1B**). Furthermore, similar results from UALCAN showed that EOGT expression was significantly upregulated in head and neck squamous cell carcinoma (HNSC), thyroid carcinoma (THCA), kidney renal clear cell carcinoma (KIRC), cholangiocarcinoma (CHOL), and HCC and was markedly downregulated in bladder urothelial carcinoma (BLCA), lung squamous cell carcinoma (LUSC), uterine corpus endometrial carcinoma (UCEC), lung adenocarcinoma (LUAD), kidney chromophobe (KICH), breast-invasive carcinoma (BRCA), and prostate adenocarcinoma (PRAD) (**Figure 1I** and **Supplementary Figure 1**).

**Figure 2:**
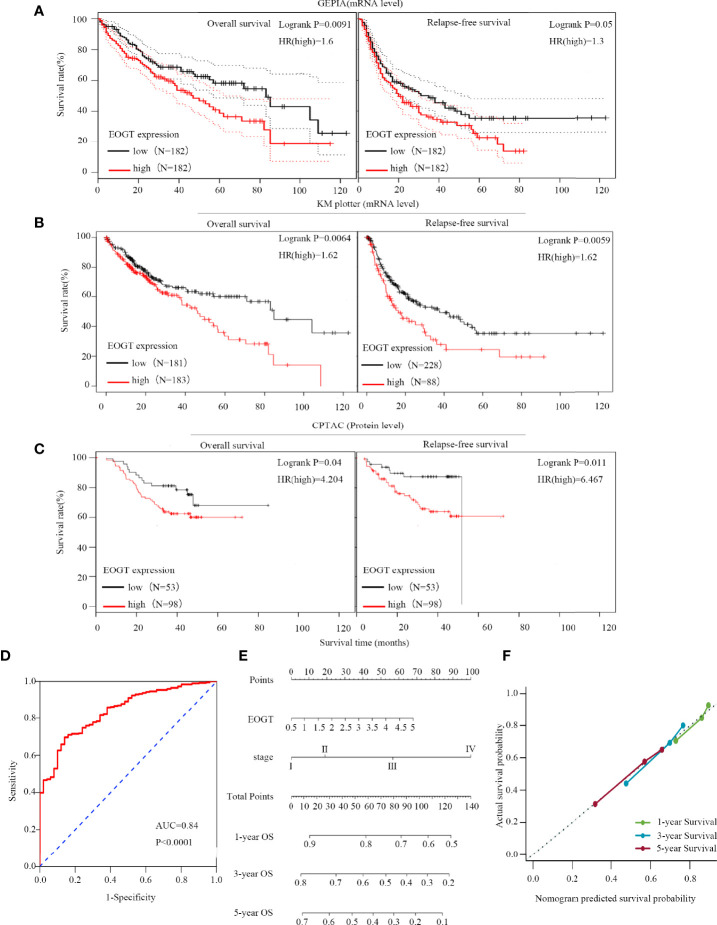
High-EOGT expression was associated with poor prognosis in HCC samples. **(A, B)** GEPIA and the Kaplan–Meier (KM) plotter were used to construct the survival curves of OS and RFS based on EOGT expression in HCC samples. **(C)** The KM survival curves based on EOGT expression at the protein level in HCC samples were determined by the CPTAC database. Validation of diagnostic value of elevated EOGT expression for HCC samples using the ROC curve **(D)**. Nomogram for HCC samples **(E)** and the calibration curve of the nomogram **(F)** for predicting OS at 1, 3, and 5 years. Actual OS was plotted on the *y*-axis; nomogram predicted probability of OS was plotted on the *x*-axis.

Since the expression of EOGT markedly transformed in multiple tumors and normal tissues, we further explored the association between EOGT expression levels and prognosis. According to EOGT mRNA expression, KM analysis was performed using GEPIA. For OS, elevated expression of EOGT only in HCC had an unfavorable prognosis. For RFS, upregulated expression of EOGT showed poor prognosis only in HCC and PRAD (**Figure 2A** and **Supplementary Figure 2**). As for the role of EOGT in predicting the survival of patients with other cancer types, no statistical significance was observed. Hence, EOGT was speculated to act as an unfavorable prognostic biomarker in HCC samples. In follow-up research, we concentrated on exploring the role of EOGT in HCC.

### High Expression of EOGT Inferred a Poor Prognosis for HCC

First of all, we further validated EOGT expression in HCC samples utilizing microarray data downloaded from GEO and Oncomine database. In line with the results of TCGA cohort, we revealed that EOGT expression was significantly upregulated in HCC samples (**Figures 1C–H**). To explore the expression of EOGT at the protein level, IHC assays performed using the HPA database were analyzed and compared the results with mRNA expression level of EOGT using UALCAN. As shown in **Figures 1I–J**, the results for IHC staining and transcriptome sequencing were in line with one another. IHC staining of EOGT was negative in normal liver samples, but positive in HCC samples. Moreover, we investigated KM analysis based on EOGT RNA expression levels *via* the KM plotter and EOGT protein expression levels *via* the CPTAC datasets. The findings supported that elevated mRNA and protein expression levels of EOGT were significantly correlated with poor OS and RFS in HCC samples (**Figures 2B, C**). For further verification, three GEO datasets (GSE54236, GSE76427, and GSE14520) with clinical data were integrated as an external validation set to further validate the prognostic value of EOGT in HCC. In the validation set, we demonstrated that EOGT expression was significantly upregulated in HCC samples (**Supplementary Figure 3I**). Besides, we also investigated survival analysis based on EOGT RNA expression levels. As shown in **Supplementary Figure 3J**, elevated RNA expression levels of EOGT were significantly correlated with poor OS in HCC, which was consistent with the above results. Taken together, EOGT expression was upregulated in HCC samples, which infers poor clinical outcomes for patients with HCC.

Besides, the ROC curve was computed to determine the diagnostic capability of EOGT for HCC. As illustrated in **Figure 2D**, AUC was found to be 0.84 (*P* < 0.0001). Moreover, in order to evaluate the independent prognostic predictor related to OS, univariate and multivariate Cox regression were implemented. The univariate analysis showed that high-EOGT expression (HR = 1.25, *P* < 0.05), tumor-node-metastasis (TNM) stage (III *vs*. I) (HR = 2.72, *P* < 0.001), TNM stage (IV vs. I) (HR = 5.44, *P* < 0.01), and vascular invasion (macro vs. none) (HR = 2.52, *P* < 0.05) significantly predicted poor OS. Plus, the multivariate analysis revealed that advanced TNM stage [TNM stage (III vs. I) (HR = 2.02, *P* < 0.05), TNM stage (IV vs. I) (HR = 5.66, *P* < 0.01)] was an independent prognostic indicator for unfavorable OS in HCC (**Table 1**). To further confirm the prognostic potential of EOGT, nomograms were performed according to the findings of multivariate Cox regression. As illustrated in **Figure 2E**, the calibration curves showed that the prediction of 1-, 3-, and 5-year OS was in excellent agreement with the actual observation. The C-index of nomogram for the prediction of OS was 0.633 (95% CI: 0.605–0.66) (**Figure 2F**). In conclusion, our results revealed that EOGT may act as a significant prognostic index in predicting OS among patients with HCC.

**Table 1:** Cox proportional hazards regression model analysis of OS.

Variables	Univariate analysis		Multivariate analysis		
	HR (95% CI)	*P*	HR (95% CI)		*P*
EOGT (high *vs*. low)	1.35 (1.01, 1.79)	**0.042**	1.25 (0.89, 1.78)		0.202
Age (≥65 *vs*. <65)	1.23 (0.85, 1.78)	0.273	–		–
Gender (female *vs*. male)	1.26 (0.87, 1.84)	0.228	–		–
Family history of cancer (yes *vs*. no)	1.14 (0.76, 1.69)	0.530	–		–
TNM stage					
(II *vs*. I)	1.42 (0.87, 2.32)	0.160			
(III *vs*. I)	2.72 (1.78, 4.15)	**<0.001**	2.02 (1.18, 3.46)		**0.011**
(IV *vs*. I)	5.44 (1.68, 17.63)	**0.005**	5.66 (1.73, 18.49)		**0.004**
Histologic grade (G3–G4 *vs*. G1–G2)	1.14 (0.78, 1.67)	0.489			
Ishak score (5–6 *vs*. 0–4)	0.82 (0.48, 1.4)	0.465	–		–
Child–Pugh grade (B–C *vs*. A)	1.66 (0.82, 3.36)	0.159	–		–
Vascular invasion					
(micro *vs*. none)	1.16 (0.72, 1.88)	0.539			
(macro *vs*. none)	2.52 (1.14, 5.58)	**0.023**	1.98 (0.87, 4.53)		0.105
Alpha fetoprotein (positive *vs*. negative)	1.45 (0.92, 2.28)	0.108	–		–
Residual tumor (R1–R2 *vs*. R0)	1.17 (0.43, 3.2)	0.754			

Statistically significant P-values are given in bold, P < 0.05.

HR, hazard ratio; CI, confidence interval.

### EOGT Correlated With Tumor Progression

**Figure 3:**
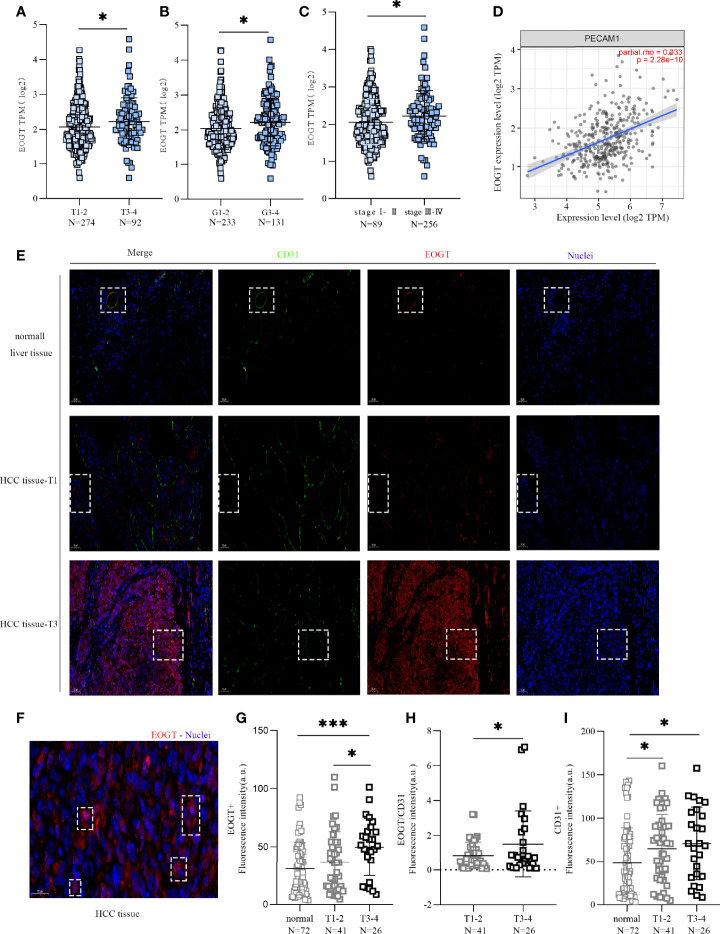
High-EOGT expression was associated with tumor staging, including TNM stage **(A)**, grade **(B)**, and stage **(C)**. **(D)** Correlation of EOGT expression with PECAM1 (CD31). **(E)** Immunofluorescent staining of EOGT (red), CD31 (green), and DAPI (blue) in normal liver tissues, T1 stage HCC tissues, and T3 stage HCC tissues. **(F)** Immunofluorescent staining of EOGT (red) and DAPI (blue) in HCC tissue. **(G)** Quantification of EOGT-positive signal in normal liver tissues (*n* = 72), T1–2 stage HCC tissues (*n* = 41), and T3–4 stage HCC tissues (*n* = 26). Values represent mean ± SEM expressed as arbitrary units (AU) of EOGT-positive area normalized by total tumor area. **(H)** Quantification of EOGT normalized by CD31 (EOGT/CD31) in T1–2 stage HCC tissues (*n* = 41) and T3–4 stage HCC tissues (*n* = 26). **(I)** Quantification of CD31-positive signal in normal liver tissues (*n* = 72), T1–2 stage HCC tissues (*n* = 41), and T3–4 stage HCC tissues (*n* = 26). Values represent mean ± SEM expressed as arbitrary units (AU) of CD31-positive area normalized by total tumor area (*P < 0.05, ***P < 0.001).

Then, we investigated the correlations between the expression level of EOGT and the progression of HCC based on TNM stage and tumor grade of the TCGA-HCC cohort. As shown in **Figures 3A–C**, HCC samples with advanced tumor staging tended to present upregulated EOGT expression. In the meantime, no significant difference between EOGT expression and vascular invasion, liver fibrosis, Child–Pugh score, or alpha fetoprotein (AFP) value was observed in HCC samples (**Supplementary Figures 3A–D**). These findings revealed that EOGT expression increased along with an increasing degree of tumor malignancy in HCC samples.

**Figure 4:**
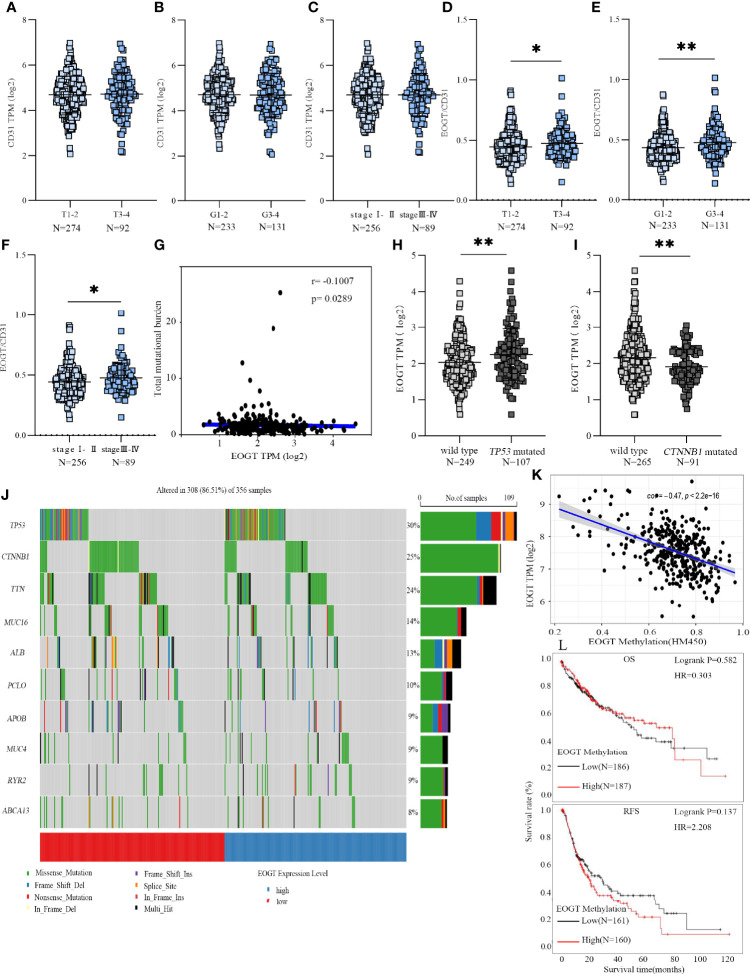
EOGT expression levels were analyzed in HCC patients with different TNM stages **(A)**, grades **(B)**, and stages **(C)**. EOGT/CD31 were analyzed in HCC patients with different TNM stages **(D)**, grades **(E)**, and stages **(F)**. Molecular characteristics of high- and low-EOGT expression subgroups; correlation analysis between EOGT expression and TMB. **(G)** Differences of EOGT expression between wild-type and *TP53* mutated subgroup **(H)** or *CTNNB1* mutated subgroup **(I)**. **(J)** Significantly mutated genes in HCC samples. Mutated genes (rows, top 10) are ordered by mutation rate. The color coding indicates the mutation of each type. **(K)** Correlation analysis between EOGT expression and EOGT DNA methylation. **(L)** KM analysis of OS and RFS based on methylation levels of EOGT (*P < 0.05, **P < 0.01).

However, a positive correlation of EOGT with tumor progression can merely be caused by aggressive angiogenesis in HCC samples because EOGT was highly expressed in endothelial cells. Therefore, we examined the correlation of PECAM1 (CD31), a pan-endothelial marker, and EOGT in HCC samples. As shown in **Figure 3D**, CD31 was significantly positively correlated with EOGT in HCC samples (Cor = 0.333, *P* < 0.0001). Nevertheless, no statistical difference between CD31 and tumor staging was observed in HCC samples (**Figures 4A–C**). We then investigated the correlations between EOGT/CD31 and HCC progression, including tumor staging, vascular invasion, liver fibrosis, Child–Pugh score, and AFP value. As shown in **Figures 4D–F**, EOGT/CD31 was obviously higher in HCC samples of advanced stages (T3–4, stage III–IV, and G3–4). Meanwhile, no significant difference was found between EOGT/CD31 and vascular invasion, liver fibrosis, or AFP value in HCC samples (**Supplementary Figures 3E–H**).

For validation, we further examined the correlations of the levels of EOGT, CD31, and EOGT/CD31 in HCC samples with T stage in TMA section. As shown in **Figure 3E**, the IF assay showed that EOGT and CD31 were co-localized in vessel structures in normal liver tissues and HCC samples. In addition, the IF assay demonstrated that EOGT protein expression was seen in the cytoplasm, nucleus, and extracellular space (**Figure 3F**). More importantly, quantitative analysis of EOGT (red) and CD31 (green) also showed that EOGT and EOGT/CD31 were obviously higher in T3–4 than in T1–2 in HCC samples (**Figures 3G, H**). Moreover, there was no statistical difference between CD31 and tumor staging (**Figure 3I**). Overall, these data presented above suggested that elevated EOGT expression may contribute to tumor development, and this process was independent of tumor angiogenesis in HCC.

### Molecular Characteristics of Different EOGT Subgroups

To gain further insight into the genetic alterations and epigenetic modifications, we firstly inquired gene mutations in both high- and low-EOGT expression subgroups. As shown in **Figure 4G**, there was no correlation between EOGT and TMB (Cor = −0.1007, *P* < 0.05). The most common variation type in **Figure 4J** was the missense mutation, followed by nonsense mutations and frameshift deletions. Subsequently, the top 10 genes with the highest mutation rates were identified in HCC samples (**Figure 4J**). The mutation rates of *TP53* and *CTNNB1* were over 25% in both high- and low-EOGT expression subgroups. Therefore, we further investigated EOGT expression between the wild-type subgroup and *TP53* or *CTNNB1* mutated subgroup. As shown in **Figures 4H, I**, the *TP53* mutation subgroup had significantly elevated EOGT expression (*P* < 0.01), whereas low-EOGT expression was found in the *CTNNB1* mutation subgroup (*P* < 0.01).

Thereafter, we investigated into the relationship between EOGT and DNA methylation. Importantly, linear regression analysis demonstrated that EOGT DNA methylation level was inversely correlated with its expression (Cor = −0.47, *P* < 0.0001) (**Figure 4K**). Moreover, survival analysis was assessed between high- and low-EOGT DNA methylation subgroups. Nevertheless, there was no statistical significance of EOGT DNA methylation levels serving as an indicator of OS (HR = 0.303, *P* = 0.582) and RFS (HR = 2.208, *P* = 0.137) (**Figure 4L**).

### Analysis of Biological Function and Construction of Protein–Protein Interaction Network

**Figure 5:**
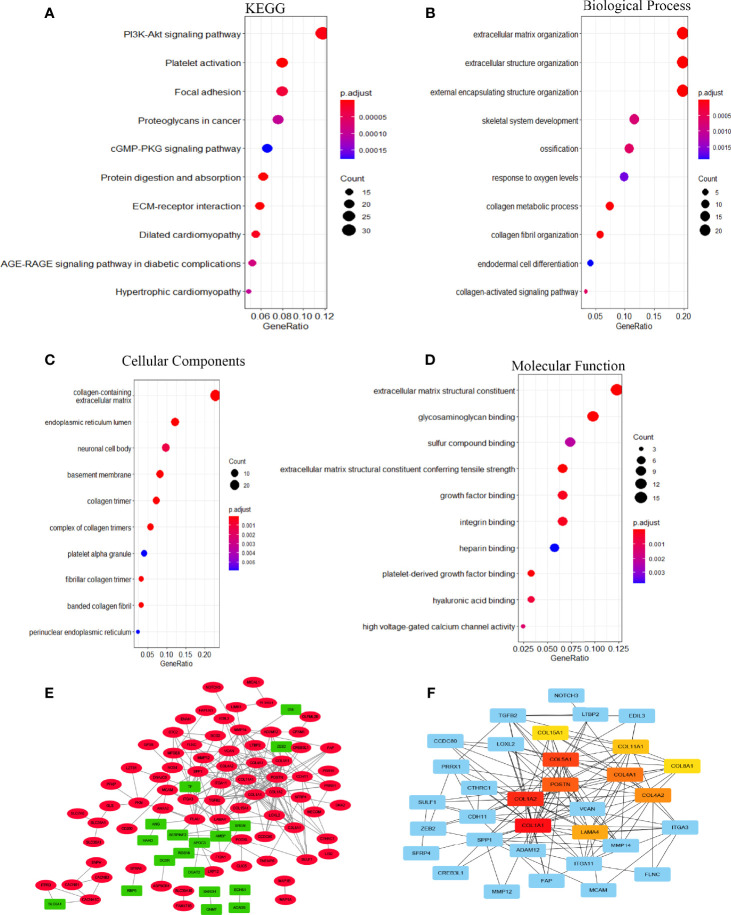
The function network of ERGs. **(A)** KEGG and **(B–D)** GO enriched terms were colored according to *P*-adjust. **(E)** Construction of gene co-expression networks. Green represented genes that were negatively related to *EOGT*, and red pointed out genes that were positively related to *EOGT*. The darker the color, the stronger the correlation. **(F)** Hub genes of the PPI network; the darker the color, the bigger the degrees.

To investigate the biological significance of EOGT in HCC samples, we performed functional enrichment analysis of ERGs, including 107 upregulated genes and 24 downregulated genes. Notably, KEGG results showed that EOGT upregulated genes were mainly enriched in the PI3K–Akt signaling pathway, focal adhesion, platelet activation, proteoglycans in cancer, cGMP–PKG signaling pathway, and so on (**Figure 5A**). For BP, these upregulated genes were primarily involved in extracellular matrix (ECM) organization, extracellular structure organization, external encapsulating structure organization, and so on (**Figure 5B**). For CC, these upregulated genes were significantly related with collagen-containing extracellular matrix, endoplasmic reticulum lumen, neuronal cell body, basement membrane, collagen trimer, and so on (**Figure 5C**). Furthermore, these upregulated genes had the MF like extracellular matrix structural constituent, glycosaminoglycan binding, sulfur compound binding, extracellular matrix structural constituent conferring tensile strength, growth factor binding, and so on (**Figure 5D**).

**Figure 6:**
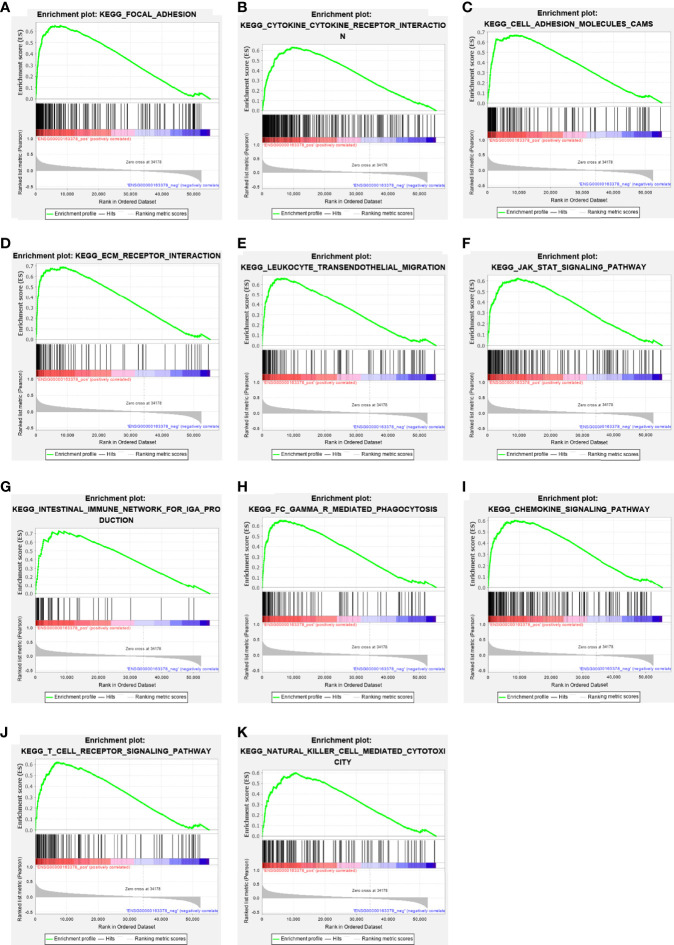
The enrichment results of GSEA. Upregulated ERGs were positively correlated with “focal adhesion” **(A)**, “cytokine–cytokine receptor interaction” **(B)**, “cell adhesion molecules” **(C)**, “ECM receptor interaction” **(D)**, “leukocyte trans-endothelial migration” **(E)**, “JAK–STAT pathway” **(F)**, “intestinal immune network for IgA production” **(G)**, “Fc gamma R-mediated phagocytosis” **(H)**, “chemokine signaling pathway” **(I)**, “T-cell receptor signaling pathway” **(J)**, and “natural killer cell-mediated cytotoxicity” **(K)**.

GSEA pathway enrichment analysis is also an effective way to elucidate EOGT biological functions. The enrichment results showed that upregulated genes were positively correlated with apoptosis, focal adhesion, cell adhesion molecules, ECM receptor interaction and cytokine–cytokine receptor interaction, JAK–STAT, chemokine, T-cell receptor, TGF-β, B-cell receptor, Toll-like receptor, MAPK and Hedgehog signaling pathways, leukocyte transendothelial migration, intestinal immune network for IgA production, Fc gamma R-mediated phagocytosis, natural killer cell-mediated cytotoxicity, and antigen processing and presentation (**Figure 6** and **Table 2**).

**Table 2:** Gene sets enriched in the high-EOGT expression phenotype.

Gene set name	ES	NES	NOM *P*-val	FDR *Q*-val
KEGG_FOCAL_ADHESION	0.65	2.56	0.000	0.000
KEGG_CYTOKINE_CYTOKINE_RECEPTOR_INTERACTION	0.63	2.55	0.000	0.000
KEGG_CELL_ADHESION_MOLECULES_CAMS	0.67	2.54	0.000	0.000
KEGG_ECM_RECEPTOR_INTERACTION	0.69	2.48	0.000	0.000
KEGG_LEUKOCYTE_TRANSENDOTHELIAL_MIGRATION	0.66	2.45	0.000	0.000
KEGG_JAK_STAT_SIGNALING_PATHWAY	0.62	2.42	0.000	0.000
KEGG_INTESTINAL_IMMUNE_NETWORK_FOR_IGA_PRODUCTION	0.73	2.39	0.000	0.000
KEGG_FC_GAMMA_R_MEDIATED_PHAGOCYTOSIS	0.65	2.39	0.000	0.000
KEGG_CHEMOKINE_SIGNALING_PATHWAY	0.60	2.36	0.000	0.000
KEGG_T_CELL_RECEPTOR_SIGNALING_PATHWAY	0.62	2.33	0.000	0.000
KEGG_NATURAL_KILLER_CELL_MEDIATED_CYTOTOXICITY	0.60	2.29	0.000	0.000
KEGG_TGF_BETA_SIGNALING_PATHWAY	0.62	2.23	0.000	0.000
KEGG_B_CELL_RECEPTOR_SIGNALING_PATHWAY	0.62	2.19	0.000	0.000
KEGG_TOLL_LIKE_RECEPTOR_SIGNALING_PATHWAY	0.56	2.07	0.000	0.000
KEGG_MAPK_SIGNALING_PATHWAY	0.51	2.04	0.000	0.000
KEGG_APOPTOSIS	0.54	1.99	0.000	0.000
KEGG_ANTIGEN_PROCESSING_AND_PRESENTATION	0.55	1.96	0.000	0.010
KEGG_HEDGEHOG_SIGNALING_PATHWAY	0.54	1.84	0.000	0.010

Gene sets with NOM P-value <0.05 and FDR Q-value ≤0.01 were considered significant. ES, enrichment score; NES, normalized enrichment score; NOM, nominal; FDR, false discovery rate.

A total of 131 genes were used to construct the PPI network which consists of 107 upregulated genes and 24 downregulated genes containing 127 nodes and 234 edges (**Figure 5E**). Ten hub genes (*COL1A1*, *COL1A2*, *COL5A1*, *POSTN*, *COL4A1*, *COL4A2*, *LAMA4*, *COL11A1*, *COL8A1*, *COL15A1*) were identified by Cytoscape based on the ranking degree calculated by CytoHubba plug-in (**Figure 5F**).

### Association Between EOGT and Various Immune Markers

**Figure 7:**
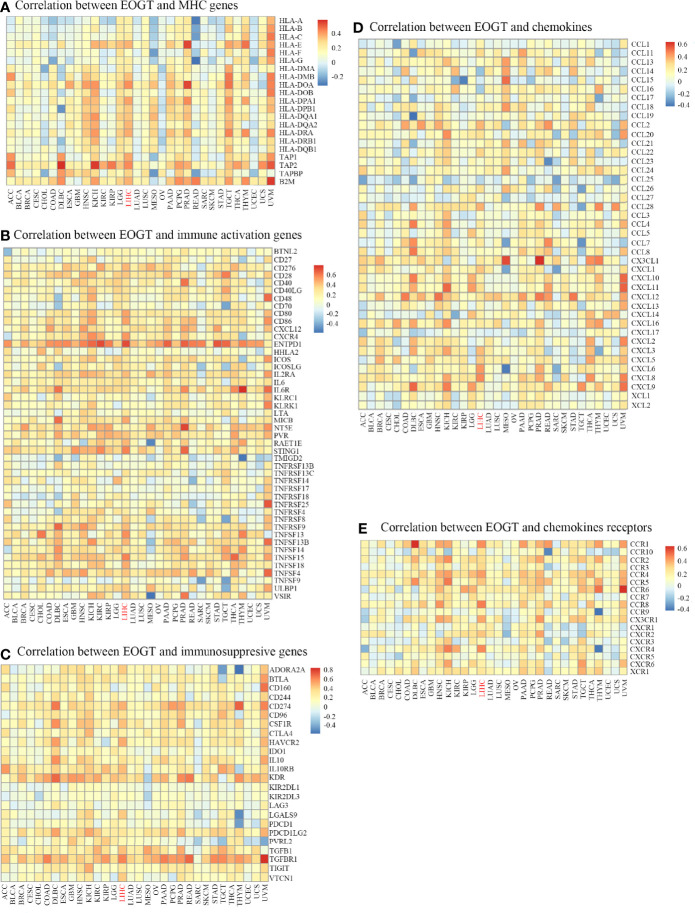
Co-expression of EOGT and immune-related genes. Correlation between EOGT and MHC genes **(A)**, Correlation between EOGT and immune activation genes **(B)**, Correlation between EOGT and immunosuppresive genes **(C)**, Correlation between EOGT and chemokines **(D)**, Correlation between EOGT and chemokines receptor **(E)**.

In the light of the above findings, we hypothesized that EOGT is involved in immune regulation in HCC. Thus, we performed correlation analyses to investigate the association between EOGT expression and a variety of IRGs. Strikingly, almost all IRGs shown in **Figure 7** were co-expressed with EOGT. Among the 150 selected genes, 131 IRGs were significantly positively correlated with EOGT in HCC tissue. In contrast, CCL14 (Cor = −0.225, *P* < 0.0001), CCL16 (Cor = −0.193, *P* < 0.001), and CCL25 (Cor = −0.155, *P* < 0.01) showed significant negative correlations with EOGT in HCC samples. Additionally, no significant relationships were observed between EOGT and TNFRSF13B, TMIGD2, CD244, KIRIDL1, LAG3, CD160, CCL15, CXCL17, CCL27, CXCL2, CCL23, XCL2, CCL19, CCL3, CCL17, and CCL13 (**Figure 7**).

To deepen our understanding of the effect of EOGT on immune regulation, we confirmed the correlations between EOGT and multiple immune biomarkers in HCC samples using GEPIA. To characterize immune cells in HCC samples, the IRGs presented in **Table 3-1** were analyzed. Our findings demonstrated that EOGT was prominently positively associated with the majority of immune markers in divergent types of immune cells. Moreover, we evaluated the correlations between EOGT and functional subsets of the various T cells. As shown in **Table 3-2**, EOGT was substantially positively associated with 30 of 34 T-cell surface markers in HCC samples.

**Table 3-1:** Correlation analysis between EOGT and biomarkers of immune cells in HCC in GEPIA2.

Immune cell	Biomarker	Cor	*P*
B cell	CD19	0.25	7.2E-07****
	CD79A	0.22	1.5E-05****
CD8^+^ T cell	CD8A	0.29	2.3E-08****
	CD8B	0.2	1.3E-04***
T cell (general)	CD3D	0.21	5.3E-05****
	CD3E	0.34	2.3E-11****
	CD2	0.31	1E-09****
Monocyte	CD86	0.54	5.9E-29****
	CD115	0.54	8.5E-29****
TAM	CCL2	0.44	5.1E-19****
	CD68	0.42	7.2E-17****
	IL10	0.45	3.8E-20****
M1 macrophage	NOS2	0.26	3.9E-07****
	IRF5	0.38	3.1E-14****
	PTGS2	0.56	2.9E-32****
M2 macrophage	CD163	0.32	4E-10****
	MS4A4A	0.5	6.6E-25****
	VSIG4	0.47	1.3E-21****
Neutrophils	CEACAM8	0.072	0.17
	ITGAM	0.55	3.6E-31****
	CCR7	0.34	2E-11****
Natural killer cell	KIR2DL1	0.15	3.4E-03**
	KIR2DL3	0.22	2.3E-05****
	KIR2DL4	0.18	4.9E-04***
	KIR3DL1	0.075	0.15
	KIR3DL2	0.24	3.3E-06****
	KIR3DL3	0.095	0.07
	KIR2DS4	0.15	5.2E-03**
Dendritic cell	HLA-DPB1	0.43	1.3E-17****
	HLA-DQB1	0.17	1.1E-03**
	HLA-DRA	0.44	1.5E-18****
	HLA-DPA1	0.46	3.5E-21****
	BCDA-1	0.3	2.5E-09****
	BDCA-4	0.53	2.2E-28****
	CD11c	0.57	5.2E-33****

Cor, R value of Spearman’s correlation; **p value < 0.01; ***p value < 0.001;****p value < 0.0001.

**Table 3-2:** Correlation analysis between EOGT and biomarkers of T cells in HCC in GEPIA2.

Immune cell	Biomarker	Cor	*P*
Th1	T-bet	0.24	2.2E-06****
	IFN-γ	0.23	5.4E-06****
	TNF-α	0.38	2.4E-14****
	STAT4	0.35	5.3E-12****
	STAT1	0.46	3.5E-21****
Th1-like	HAVCR2	0.54	1.6E-29****
	CXCR3	0.29	2.1E-08****
	BHLHE40	0.4	2.5E-15****
	CD4	0.41	1.3E-16****
Th2	GATA3	0.43	6E-18****
	STAT5A	0.51	1.8E-25****
	STAT6	0.45	1.4E-19****
	IL13	0.08	0.13
Treg	FOXP3	0.23	1.2E-05****
	STAT5B	0.43	2E-18****
	TGFβ	0.43	2.2E-18****
	CCR8	0.53	1E-27****
Resting Treg	FOXP3	0.23	1.2E-05****
	IL2RA	0.47	5.3E-22****
Effector Treg	FOXP3	0.23	1.2E-05****
	CCR8	0.53	1E-27****
	TNFRSF9	0.39	9.9E-15****
Effector T cell	CX3CR1	0.46	1E-20****
	FGFBP2	−0.062	0.24
	FCGR3A	0.46	8.4E-21****
Naive T cell	CCR7	0.34	2E-11****
	SELL	0.44	4.9E-19****
Effector memory T cell	DUSP4	0.51	2.4E-25****
	GZMK	0.28	3.8E-08****
	GZMA	0.22	1.8E-05****
Resident memory T cell	CD69	0.43	8.5E-18****
	CXCR6	0.34	3.1E-11****
	MYADM	0.53	3.6E-28****
General memory T cell	CCR7	0.34	2E-11****
	SELL	0.44	4.9E-19****
	IL7R	0.43	9.2E-18****
Tfh	BCL6	0.3	4.4E-09****
	IL21	0.096	0.065
Th17	STAT3	0.51	8.2E-26****
	IL17A	0.047	0.37

Cor, R value of Spearman’s correlation;****p value < 0.0001.

### EOGT Positively Correlated With Immunosuppressive TME in HCC

**Figure 8:**
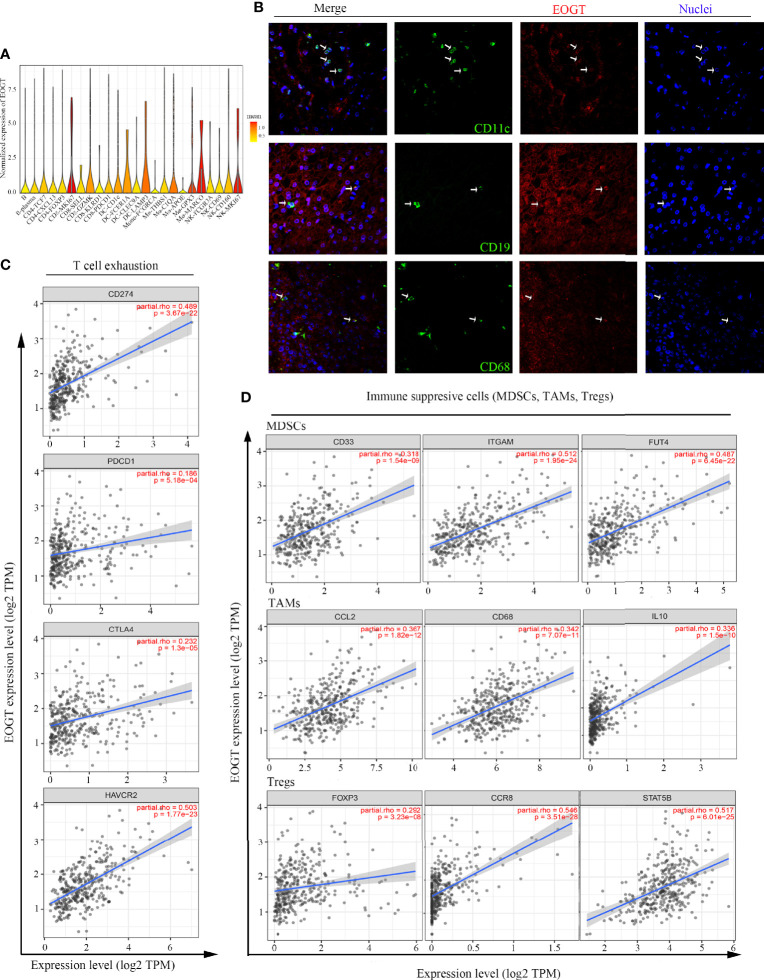
Analysis of immune cell infiltration in HCC. **(A)** EOGT expression was highly expressed on different immune cell subsets according to single-cell RNA-sequencing results of six HCC patients. **(B)** Immunofluorescent staining of EOGT (red), CD11c (green), CD19 (green), CD68 (green), and DAPI (blue) in HCC tissue. **(C)** Correlations of EOGT expression with immunosuppressive molecules (PD-L1, PD-1, CTLA4, and HAVCR2) involved in T-cell exhaustion. **(D)** Correlations of EOGT expression with markers of immunosuppressive cells (MDSCs, TAMs, and Tregs).

**Figure 9:**
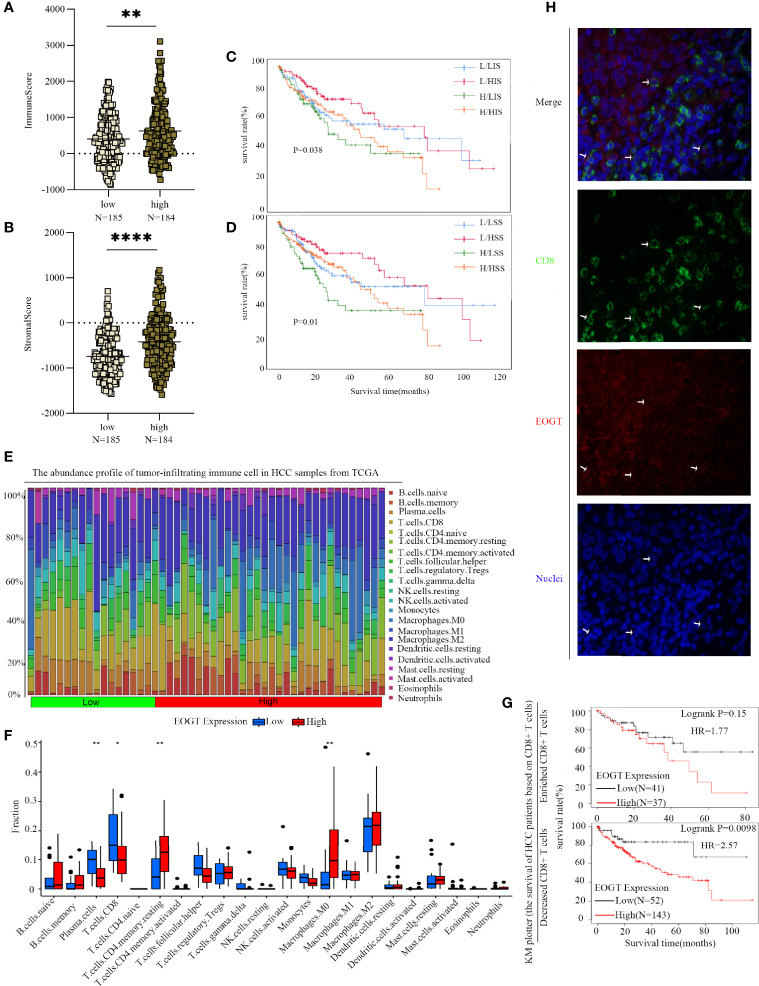
Differences of immune scores **(A)** and stromal scores **(B)** between EOGT-low and EOGT-high expression subgroups. **(C, D)** KM survival curve of OS based on immune scores, stromal scores, and EOGT expression levels. L and H, respectively, represented the low-EOGT and high-EOGT expression groups. LIS and HIS, respectively, represented low-immune scores group and high-immune scores group. LSS and HSS, respectively, represented low-stromal scores group and high-stromal scores group. **(E)** Twenty-two tumor-infiltrating immune cells in HCC samples were estimated using the CIBERSORT algorithm. **(F)** The proportion of 22 tumor-infiltrating immune cells in HCC samples with high- and low-EOGT expression. **(G)** KM curves of OS in HCC samples based on EOGT expression and the levels of tumor-infiltrating CD8^+^ T cells. **(H)** Immunofluorescent staining of EOGT (red), CD8 (green), and DAPI (blue) in HCC tissues (*P < 0.05, **P < 0.01, ****P < 0.0001).

The immune infiltration within the TME was closely related to the occurrence and development of HCC. To further refine the association between EOGT and tumor-infiltrating immune cells, we analyzed single-cell RNA-sequencing data to interrogate the relationship. As shown in **Figure 8A**, we found that EOGT was highly expressed on B cells, CD4^+^ T cells, CD8^+^ T cells, DCs, macrophages, and NK cells. For validation, we performed double-labeling IF staining with EOGT (red) and CD19, CD4, CD8, CD11c, CD68, or CD56 (green) antibodies. IF staining results showed that EOGT was positive on CD11c^+^ cells, CD19^+^ cells, CD68^+^ cells, and CD8^+^ T cells (**Figures 8B**, **9H**), but negative on CD4^+^ cells and CD56^+^ cells (data not shown).

In addition, we utilized TIMER to explore the relationships between EOGT and immunosuppressive molecules and cells. The analysis presented that EOGT was positively correlated with programmed death ligand 1 (PD-L1; Cor = 0.489, *P* < 0.0001), hepatitis A virus cellular receptor 2 (HAVCR2; Cor=0.503, *P* < 0.0001), programmed cell death protein 1 (PD-1; Cor = 0.186, *P* < 0.001), and cytotoxic T lymphocyte-associated antigen-4 (CTLA4; Cor = 0.232, *P* < 0.0001) (**Figure 8C**). Moreover, immune markers of main immunosuppressive cells in HCC including myeloid-derived suppressor cells (MDSCs) (CD33: Cor = 0.318, *P* < 0.0001; ITGAM: Cor = 0.512, *P* < 0.0001; FUT4: Cor = 0.487, *P* < 0.0001), TAMs (CCL2: Cor = 0.367, *P* < 0.0001; CD68: Cor = 0.342, *P* < 0.0001; IL-10: Cor = 0.336, *P* < 0.0001), and Tregs (FOXP3: Cor = 0.292, *P* < 0.0001; CCR8: Cor = 0.546, *P* < 0.0001; STAT5B: Cor = 0.517, *P* < 0.0001) were positively associated with EOGT (**Figure 8D**). Taken together, our findings revealed that EOGT was closely associated with the immunosuppressive TME in HCC.

### Poor Prognosis of HCC Patients Was Partly due to Reduced Infiltration of CD8^+^ T Cells Caused by Elevated Expression of EOGT

To further clarify the interaction of EOGT with immunosuppressive TME in HCC, on one hand, the relationships between EOGT and stromal or immune scores were examined using the ESTIMATE method. Our results revealed that samples with elevated EOGT expression had obviously increased immune scores (*P* < 0.01) as well as stromal scores (*P* < 0.0001) (**Figures 9A, B**). To investigate the association of stromal and immune scores with prognosis, HCC samples were classified into high- and low-score subgroups according to the median of immune and stromal scores, respectively. As shown in **Figure 9C**, patients in the elevated EOGT expression and low-immune scores subgroup had a lower OS than low-EOGT expression and high-immune scores subgroup. Patients in the high-EOGT expression and low-stromal scores subgroup had a lower OS than low-EOGT expression and high-stromal scores subgroup (**Figure 9D**).

On the other hand, we explored the relationships between EOGT and 22 types of tumor-infiltrating immune cells in HCC using CIBERSORT algorithm (**Figure 9E**). The analysis revealed that HCC samples with elevated EOGT expression had markedly low fractions of plasma cells (*P* < 0.01) and CD8^+^ T cells (*P* < 0.05), whereas HCC samples with elevated EOGT expression had significantly high fractions of CD4^+^ memory resting T cells (*P* < 0.01) and M0 macrophages (*P* < 0.01). Nevertheless, there was no significant difference detected in the infiltration of other immune cells between high- and low-EOGT expression subgroups (**Figure 9F**). Since EOGT was obviously linked to immunosuppressive TME and poor outcomes in HCC samples, we explored whether EOGT affected the prognosis of patients with HCC through acting on TME. Subgroup analysis revealed that elevated EOGT expression with enriched Tregs indicated poor outcomes in samples with reduced CD8^+^ T cells, but not in those with enriched CD8^+^ T cells (**Figure 9G**).

This observation was supported by external validation below. The results based on the validation set revealed that HCC samples with elevated EOGT expression had markedly low fractions of naive B cells (*P* < 0.01), CD8^+^ T cells (*P* < 0.001), and monocytes (*P* < 0.001), whereas HCC samples with elevated EOGT expression had significantly high fractions of T follicular helper cells (*P* < 0.001), M0 macrophages (*P* < 0.001), and neutrophils (*P* < 0.01) (**Supplementary Figure 3K**). Besides, similar to previous results in **Figure 9G**, elevated expression of EOGT indicated poor outcomes in samples with reduced CD8^+^ T cells, but not in those with enriched CD8^+^ T cells (**Supplementary Figure 3L**). Collectively, the above findings revealed that elevated EOGT expression in HCC samples facilitated tumor development and poor outcomes at least partly due to the reduced number of CD8^+^ T cells.

## Discussion

ICI therapy has proven to be an effective treatment for advanced HCC patients. Given that the reported response rate to ICIs in advanced HCC was quite low, the identification of patients who will derive durable benefit from immunotherapies is critical (13). In recent years, a variety of prognostic markers has been explored in HCC, but well-validated biomarkers have not been found for predicting the response to ICI treatment and prognosis. This emphasized the importance of establishing a prognostic biomarker for immunotherapy in HCC.

Aberrant glycosylation has been intricately linked with the development of cancer and, more recently, with tumor immunogenicity (14). There are two major types of glycosylation, namely, N-glycosylation and O-glycosylation. Increasing lines of evidence suggested that heavy N-linked glycosylation of PD-L1 maintained its interaction with PD-1, facilitating the evasion of T-cell-mediated immunity (15). In addition, it has been well shown that mucins were highly glycosylated with O-linked oligosaccharides and multiple mucin domains interacted with crucial components of TME, which correlated with the role of O-glycosylation in tumor immunity. However, the precise interaction implicated in O-glycosylation has not yet been elucidated. Here, we focused on the role of EOGT in HCC and sought to elucidate the role of O-glycosylation on tumor immune infiltration in HCC.

First of all, our pan-cancer analysis showed that EOGT was highly expressed in five types of cancer, namely, CHOL, HNSC, KIRC, HCC, and THCA. However, there were studies that showed that EOGT expression was increased in PAAD (9, 10), which contradicted with our results, because they did not consider the classical subtype of PAAD. Results from GEO and Oncomine consistently demonstrated that EOGT expression was obviously elevated in HCC samples. Moreover, regarding survival analysis, high-EOGT expression only served as an unfavorable prognostic indicator of both OS and RFS in HCC, but not for any other cancers. Notably, KM survival analysis using the KM plotter and CPTAC further proved that elevated EOGT expression was closely linked to poor OS and RFS in HCC samples. Moreover, it was reported that low expression of both EOGT and LFNG was associated with favorable OS in pancreatic ductal adenocarcinoma patients (9). In a recent study, IHC staining in sequencing samples confirmed that EOGT was linked to poor OS in pancreatic cancer (10). To further understand the role of EOGT in the progression of HCC, we explored the relationship between tumor staging and the prognostic value of EOGT. Initially, we discovered that EOGT was upregulated in patients with advanced tumor staging. Furthermore, ROC curves showed that EOGT exhibited excellent diagnostic efficiency for HCC. Meanwhile, multivariate analyses and nomogram also confirmed that elevated EOGT was a significant indicator of unfavorable OS in HCC. These data indicated that EOGT may be a promising prognostic indicator for advanced HCC patients.

To provide more comprehensive insight into the signature of EOGT, we identified gene mutations associated with the expression levels of EOGT. Recent whole-genome sequencing revealed that mutations in *TP53* and its related molecules, such as *CTNNB1*, *AXIN1*, and *BRD7*, define core pathways that are commonly deregulated in HCC (16). Our data have shown that the main difference in mutations between high- and low-EOGT expression were in *TP53* and *CTNNB1* mutations. Notably, our data showed that *TP53* mutation, the most common genetic alternations in tumorigenesis, occurred more frequently in high-EOGT expression subgroup than in low-EOGT expression subgroup, contributing to tumor invasiveness and poor outcomes in multiple cancer types (17), particularly in HCC (18). Additionally, a previous study has demonstrated that *CTNNB1* mutation subgroup was significantly associated with a better prognosis and a higher TMB compared with the wild-type subgroup in the TCGA-HCC cohort (19). Surprisingly, there was a higher mutation rate of *CTNNB1* in low-EOGT subgroup than in the high-EOGT subgroup, which implied that the low-EOGT subgroup might be associated with a better prognosis, in agreement with our survival results. In conclusion, the elevated EOGT expression subgroup with high *TP53* and low *CTNNB1* mutations had a worse prognosis than the low-EOGT expression subgroup with low *TP53* and high *CTNNB1* mutations.

In the present study, GO enrichment analyses of ERGs revealed that EOGT was closely associated with the assembly, arrangement, or disassembly of the ECM. On the side, KEGG pathway enrichment analyses and GSEA results of ERGs exhibited that EOGT was implicated in various pathways, especially immune-related pathways, including cytokine–cytokine receptor interaction, ECM receptor interaction, leukocyte transendothelial migration, and JAK–STAT signaling pathway in HCC. Through PPI network construction, key hub genes were identified, including *COL1A1*, *COL1A2*, *COL5A1*, *POSTN*, *COL4A1*, *COL4A2*, *LAMA4*, *COL11A1*, *COL8A1*, and *COL15A1*, which had a completely positive correlation with *EOGT*. Interestingly, some researches have shown that COL1A2, COL1A1, COL5A1, and POSTN were identified to be involved in TMB and immune infiltration, which were linked to poor outcomes of clear cell renal cell carcinoma (20). Moreover, COL1A1 was involved in the progression of a variety of cancer types, such as gastric cancer and pancreatic cancer (21–23). Research has shown that promising capabilities of COL1A1 predict immunotherapy response in GC patients (24). Additionally, some studies showed that COL1A1, COL1A2, and COL4A1could regulate the immunosuppressive TME of glioma (25). Furthermore, it was indicated that highly expressed COL4A2 was positively associated with decreased survival as well as infiltration of macrophages and DCs in patients with cervical cell carcinoma. Meanwhile, a study of malignant melanoma found that mutations in COL5A1 were linked to the infiltration of CD8^+^ T cells and activated NK cells. Among them, Laminin alpha 4 (LAMA4) was associated with outcomes and immune infiltration in GC, including CD4^+^ T cells, DCs, and TAMs (26). In summary, the above results may indicate that EOGT has been strongly implicated in tumor immunity.

To confirm the correlation between EOGT and tumor immunity, heatmaps suggested that CCL14 was significantly negatively correlated with EOGT in HCC. It has been documented that CCL14 was decreased in HCC samples relative to normal samples, and low expression of CCL14 in HCC samples was linked to unfavorable outcomes (27). Plus, some studies have proposed that the expression of CCL14 in HCC was negatively correlated with the expression of exhausted T-cell markers (28). This can be partly explained by the fact that the biological activity of CCL14 was regulated by glycosylation (29). As described above, EOGT was significantly correlated with immune cell infiltration and impacted clinical outcomes. The reduced tumor-infiltrating CD8^+^ T cells led to unfavorable prognosis and impaired immune regulation against HCC development (30). Our results then presented that when EOGT was highly expressed in HCC, the number of CD8^+^ T cells significantly reduced. Besides, the prognostic significance of EOGT was not found in HCC samples with enriched CD8^+^ T cell. Hence, there were reasons to infer that upregulated EOGT expression attributed to unfavorable outcomes and HCC development through inhibiting the infiltration of CD8^+^ T cells. As it is known to us, TME signatures could serve as effective biomarkers to evaluate immunotherapy response and affect prognosis (31). The results of the ESTIMATE algorithm showed that the highly expressed EOGT subgroup displayed higher immune score and stromal score compared with the low-EOGT expression subgroup in HCC. In addition, low immune score and stromal score with the high-EOGT expression subgroup indicated poorer OS than the other groups. A previous study suggested that immune score and stromal score were significantly correlated with poor outcomes in HCC, which was consistent with our findings (19).

Here, we found, for the first time, positive correlations of EOGT expression with the infiltration of immunosuppressive cells in HCC samples, including MDSCs, TAMs, and Tregs. It is generally known that the TME was in an immunosuppressive state associated with a severe dysregulation of the immune response through numerous mechanisms, including accumulation of abundant immunosuppressive cytokines, presence of immunosuppressive cells, and exhaustion of T cells that interacted with immune checkpoint receptors (32). As previous literature reported, the upregulation of inhibitory receptors such as PD-1, PD-L1, CTLA-4, and HAVCR2 has been observed in exhausted T cells (33). These studies were in agreement with our results. In conclusion, these results suggested that EOGT could be a promising immune-related therapeutic biomarker in HCC.

This study systematically characterizes the interplay between EOGT and tumor immune infiltration in HCC, but there still exist some limitations in our project. Initially, the detailed molecular mechanisms regarding how EOGT regulates immunosuppressive TME still need further studies to elucidate. Besides, further in-depth exploration is needed to verify its clinical significance in guiding immunotherapy. In summary, EOGT was a promising immune-related prognostic biomarker and may help in distinguishing immune and molecular characteristics, predicting outcomes in HCC patients.

## Data Availability Statement

The datasets presented in this study can be found in online repositories. The names of the repository/repositories and accession number(s) can be found in the article/**Supplementary Material**.

## Ethics Statement

The studies involving human participants were reviewed and approved by the Ethics Committee of Beijing Ditan Hospital of Capital Medical University for the protection of human subjects. The patients/participants provided their written informed consent to participate in this study.

## Author Contributions

HSW conceived the study. YS drafted the manuscript and performed the analysis. LH, MG, FX, JY, SW, HRW, and FZ assisted in performing the literature search and collecting data. All authors contributed to the article and approved the submitted version.

## Funding

This work was financially supported by the National Natural Science Foundation of China (82170541), Digestive Medical Coordinated Development Center of Beijing Hospitals Authority (No. XXZ0404), Capital Foundation for Clinical Characteristic Applied Research Projects (Z181100001718084), Beijing Natural Science Foundation (7202071), and the Study on Modernization of Traditional Chinese Medicine (2018YFC1705700).

## Conflict of Interest

The authors declare that the research was conducted in the absence of any commercial or financial relationships that could be construed as a potential conflict of interest.

## Publisher’s Note

All claims expressed in this article are solely those of the authors and do not necessarily represent those of their affiliated organizations, or those of the publisher, the editors and the reviewers. Any product that may be evaluated in this article, or claim that may be made by its manufacturer, is not guaranteed or endorsed by the publisher.
