# Supplementary material for: EOGT Correlated With Immune Infiltration: A Candidate Prognostic Biomarker for Hepatocellular Carcinoma
Source: Front Immunol. 2022 Jan 5;12:780509. doi: 10.3389/fimmu.2021.780509 (PMC8766744; doi:10.3389/fimmu.2021.780509)
Supplement: Supplementary file 1 [file DataSheet_1.pdf]

Supplementary Material

1 Supplementary Figures

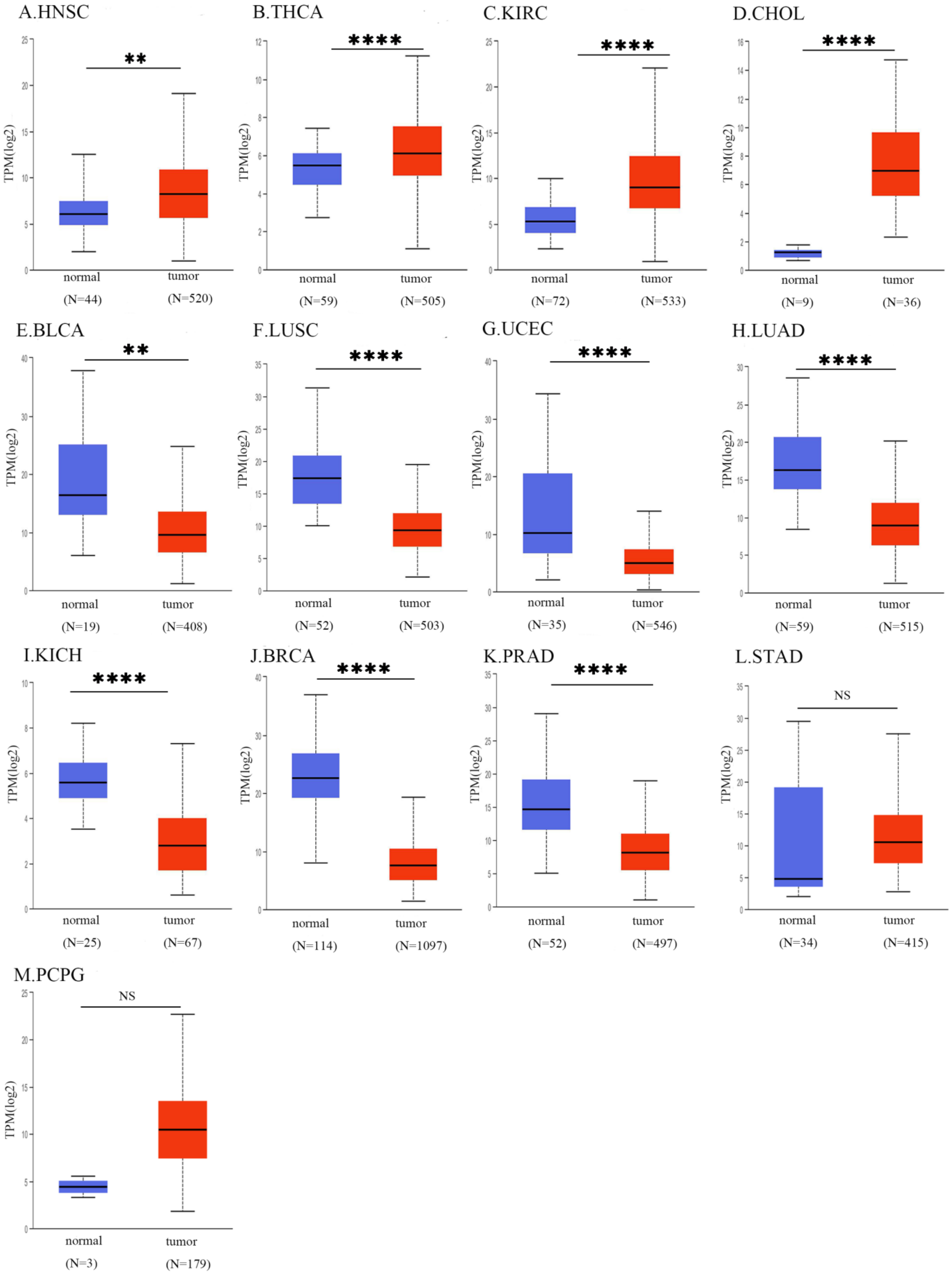
A.HNSC
**
TPM(log2)
normal (N=44)
tumor (N=520)
B.THCA
****
TPM(log2)
normal (N=59)
tumor (N=505)
C.KIRC
****
TPM(log2)
normal (N=72)
tumor (N=533)
D.CHOL
****
TPM(log2)
normal (N=9)
tumor (N=36)
E.BLCA
**
TPM(log2)
normal (N=19)
tumor (N=408)
F.LUSC
****
TPM(log2)
normal (N=52)
tumor (N=503)
G.UCEC
****
TPM(log2)
normal (N=35)
tumor (N=546)
H.LUAD
****
TPM(log2)
normal (N=59)
tumor (N=515)
I.KICH
****
TPM(log2)
normal (N=25)
tumor (N=67)
J.BRCA
****
TPM(log2)
normal (N=114)
tumor (N=1097)
K.PRAD
****
TPM(log2)
normal (N=52)
tumor (N=497)
L.STAD
NS
TPM(log2)
normal (N=34)
tumor (N=415)
M.PCPG
NS
TPM(log2)
normal (N=3)
tumor (N=179)

Supplementary Figure 1. Expression analysis for EOGT in multiple cancers (A–M) EOGT expression in TCGA HNSC (A), THCA (B), KIRC (C), CHOL (D), BLCA (E), LUSC (F), UCEC (G) LUAD (H), KICH (I), BRCA (J), PRAD (K), STAD (L) and PCPG (M) tissues compared with corresponding TCGA normal tissues. (**P <0 .01, ****P <0.0001)

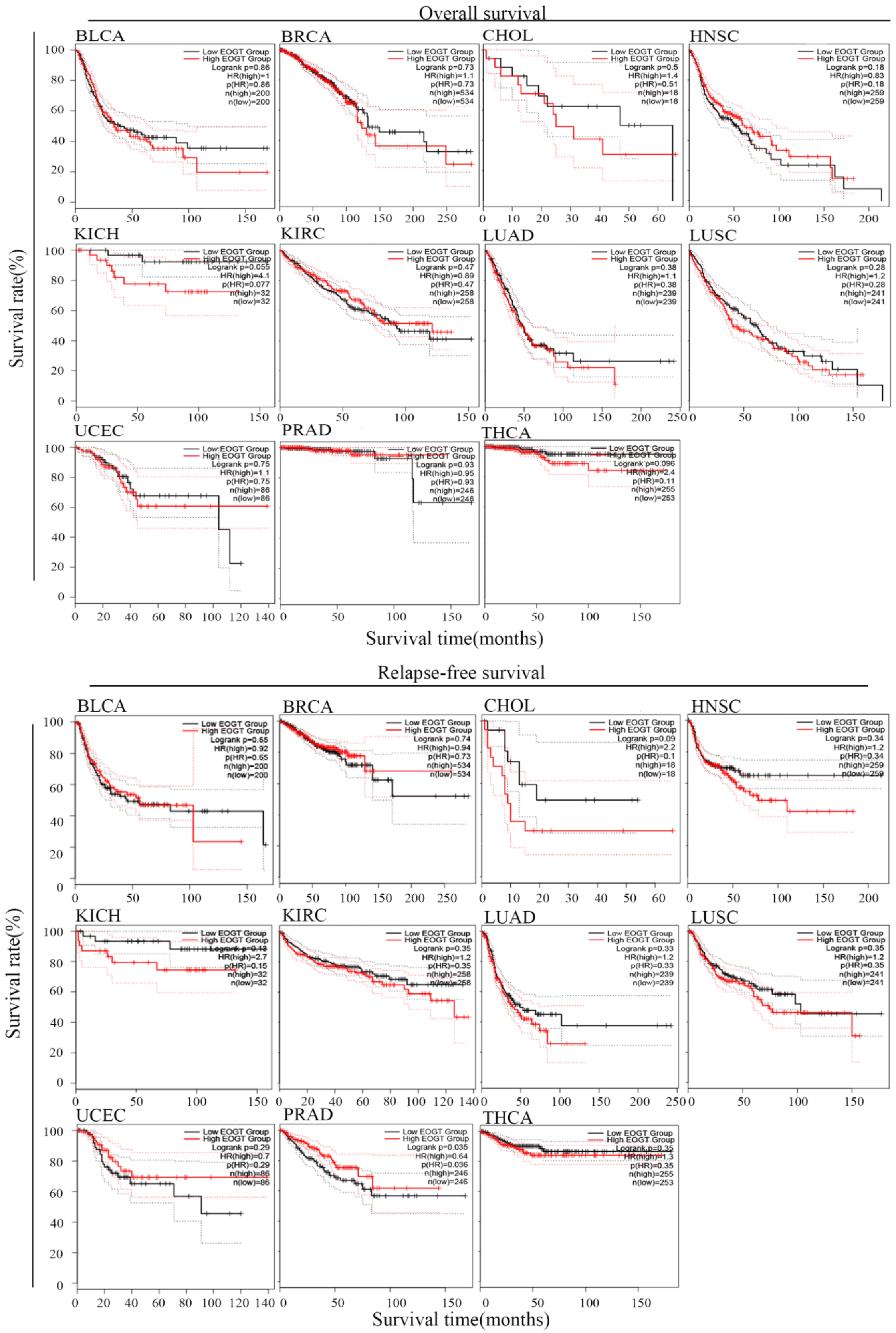
Overall survival
BLCA
BRCA
CHOL
HNSC
KICH
KIRC
LUAD
LUSC
UCEC
PRAD
THCA
Survival rate(%)
Survival time(months)
Relapse-free survival
BLCA
BRCA
CHOL
HNSC
KICH
KIRC
LUAD
LUSC
UCEC
PRAD
THCA
Survival rate(%)
Survival time(months)

Supplementary Figure 2. KM survival analysis of the association between EOGT expression and OS and RFS.

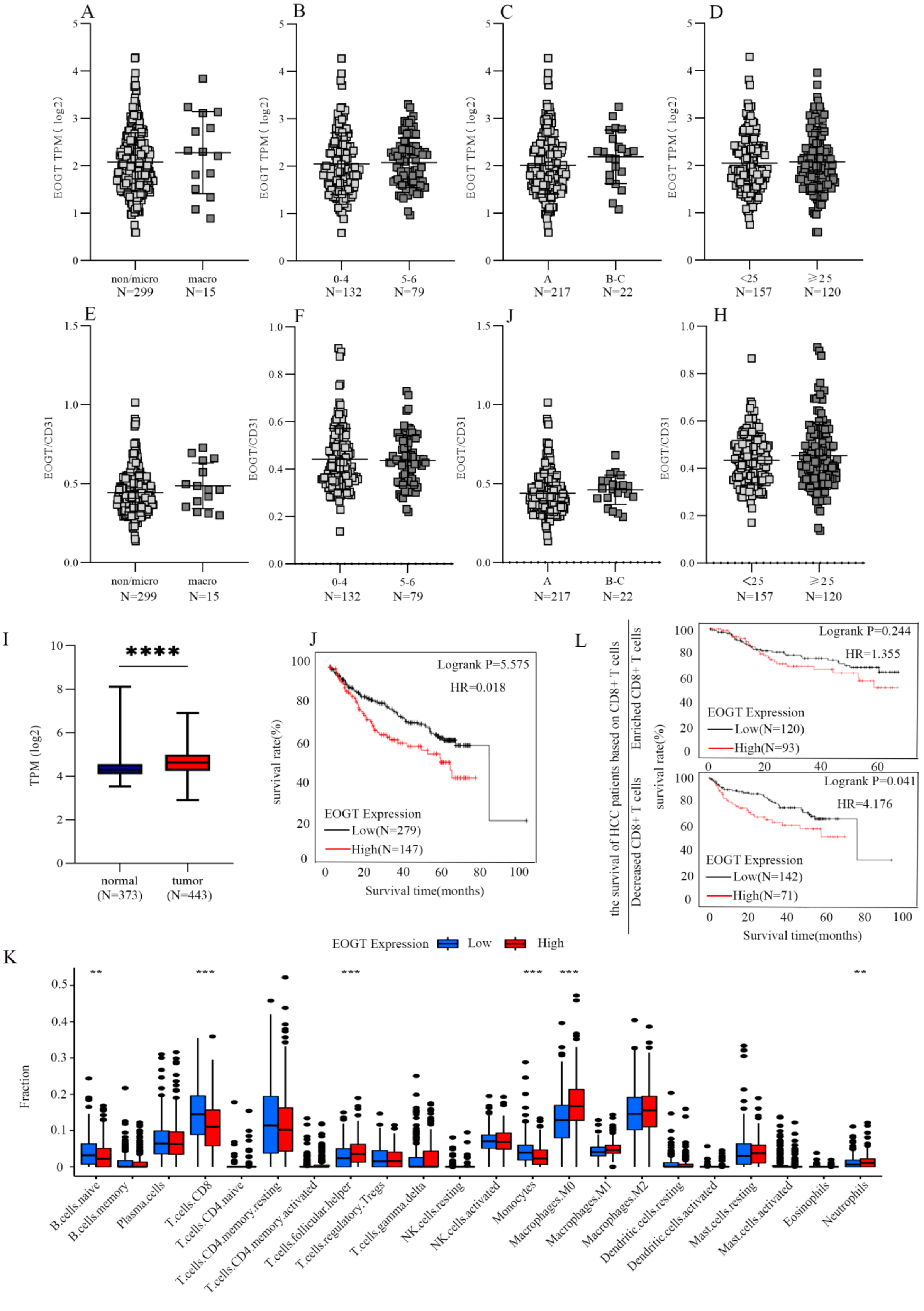

A
B
C
D
EOGT TPM (log2)
non/micro N=299
macro N=15
0-4 N=132
5-6 N=79
A N=217
B-C N=22
<25 N=157
⩾25 N=120
E
F
J
H
EOGT/CD31
I
TPM (log2)
****
normal (N=373)
tumor (N=443)
J
Logrank P=5.575
HR=0.018
survival rate(%)
EOGT Expression
Low(N=279)
High(N=147)
Survival time(months)
L
the survival of HCC patients based on CD8+ T cells
Enriched CD8+ T cells
Decreased CD8+ T cells
Logrank P=0.244
HR=1.355
Low(N=120)
High(N=93)
Logrank P=0.041
HR=4.176
Low(N=142)
High(N=71)
K
EOGT Expression Low High
Fraction
B.cells.naive
B.cells.memory
Plasma.cells
T.cells.CD8
T.cells.CD4.naive
T.cells.CD4.memory.resting
T.cells.CD4.memory.activated
T.cells.follicular.helper
T.cells.regulatory.Tregs
T.cells.gamma.delta
NK.cells.resting
NK.cells.activated
Monocytes
Macrophages.M0
Macrophages.M1
Macrophages.M2
Dendritic.cells.resting
Dendritic.cells.activated
Mast.cells.resting
Mast.cells.activated
Eosinophils
Neutrophils

Supplementary Figure 3. Correlations between EOGT expression and clinicopathological characteristics, including vascular invasion (A), liver fibrosis (B), Child-Pugh score (C), and AFP value (D). Correlations between EOGT/CD31 and clinicopathological characteristics, including vascular invasion (E), liver fibrosis (F), Child-Pugh score (G), and AFP value (H). (I) The expression level of EOGT in normal liver and HCC tissues from validation set. (J) Survival analysis based on the expression levels of EOGT from validation set. (K) The proportion of 22 tumor-infiltrating immune cells in HCC samples from validation set with high and low EOGT expression. (L) KM curves of OS in HCC samples from validation set based on EOGT expression and the levels of tumor-infiltrating CD8+ T cells. (**P <0 .01, ***P <0 .001, ****P <0.0001)
